# First molecular approach to the octopus fauna from the southern Caribbean

**DOI:** 10.7717/peerj.7300

**Published:** 2019-07-29

**Authors:** Elena A. Ritschard, Jürgen Guerrero-Kommritz, Juan A. Sanchez

**Affiliations:** 1 Laboratorio de Biología Molecular Marina (BIOMMAR), Departamento de Ciencias Biológicas- Facultad de Ciencias, Universidad de Los Andes, Bogotá, Colombia; 2 Department of Molecular Evolution and Development, University of Vienna, Vienna, Austria; 3 Fundación Fundabas, Bogotá, Colombia

**Keywords:** Octopodidae, Molecular phylogeny, *Rhodopsin*, Southern Caribbean, Species delimitation, Octopus, *Cytochrome c oxidase subunit I*

## Abstract

The octopus fauna from the southern Caribbean is an understudied field. However, recent taxonomic work in the Colombian Caribbean has led to the discovery of several new species in the family Octopodidae. To provide molecular evidence for recent descriptions in the area (i.e., *Octopus taganga, O. tayrona* and *Macrotritopus beatrixi*) and contribute to the systematics of the family, we reconstructed the first molecular phylogenies of the family including Colombian Caribbean octopus species. Using *cytochrome c oxidase subunit I* and *rhodopsin* sequences from specimens collected in three sites (Santa Marta, Old Providence and San Andrés Islands) we inferred maximum-likelihood trees and delimited species with PTP. Our mitochondrial analysis supported the monophyly of species found in the area (i.e., *O. taganga*, *O. hummelincki* and *O. briareus*). The genetic distinction of the species *O. tayrona* and *O. insularis* was not resolved, as these were found in one clade together with Caribbean *O. vulgaris* and *O.* aff*. tayrona* species (*O.* spB) and delimited as a single species. Additionally, our results suggest a distant relationship of the Type I *O. vulgaris* group (Caribbean region) from the other forms of the species complex (Old World and Brazil). Lastly, the third newly described species *M. beatrixi* emerged as an independent lineage and was delimited as a single species. However, its relationship to other species of its genus remains unknown due to the lack of sequences in databases. Altogether, our molecular approach to the octopus fauna from the southern Caribbean adds on information to the relationship of Octopodidae species world-wide by providing sequences from recently described species from an understudied region. Further studies employing higher taxon sampling and more molecular information are needed to fill taxonomic gaps in the area and account for single-locus resolution on the systematics of this group.

## Introduction

The Caribbean region represents a global-scale hotspot of marine biodiversity, which includes the greatest concentration of marine species in the tropical Atlantic Ocean (Roberts et al., 2002). However, many gaps in biodiversity studies remain and need to be filled in order to define conservation priorities and design regional-scale management strategies (Miloslavich et al., 2010). An accurate evaluation of marine biodiversity in the Caribbean needs comprehensive identification guides and taxonomic expertise. This phenomenon has also been called *taxonomic impediment* (see Wheeler, 2004; Wheeler, Raven & Wilson, 2004; Crisci, 2006) and, together with poor sampling efforts, represents an enduring issue for many invertebrates (e.g*.,* Coleman, 2015), including cephalopods (Norman & Hochberg, 2005).

All of the major cephalopod lineages contain taxa with unstable systematics (Allcock, Strugnell & Johnson, 2008). In 2005, Norman and Hochberg reported 186 resolved octopus species names in the family Octopodidae d’Orbigny. Until then, around 50% of the described species were lumped in the genus *Octopus* Cuvier, 1798 (Allcock, Strugnell & Johnson, 2008). This genus, *sensu stricto,* has only 11 accepted species, all other belong to different genera and most still need to be defined (Norman, Finn & Hochberg, 2014). This has led to an underestimation of several genera within Octopodidae. Regional surveys around the globe additionally revealed a higher octopod diversity, with a recent increase in discovery of new species (Norman & Hochberg, 2005). Moreover, in recent years, studies of cryptic species complexes have increased the number of putative species in the family (e.g., Amor et al., 2016; Amor et al., 2014; Leite et al., 2008). Despite these improvements to octopod systematics, some regions around the globe remain understudied. For instance, within the highly diverse Caribbean, its southern area (Panama, Venezuela and Colombia) represents a remarkable taxonomic gap of the Octopodidae (Guerrero-Kommritz & Camelo-Guarin, 2016).

Twenty-nine (29) cephalopod species have been reported from the Colombian Caribbean (Díaz, Ardila & Gracia, 2002; Díaz, Ardila & Gracia, 2000; Guerrero-Kommritz & Camelo-Guarin, 2016; Guerrero-Kommritz et al., 2016) and 12 of them belong to the family Octopodidae *sensu* Strugnell et al. (2014). Recent taxonomic work based on morphology (Guerrero-Kommritz & Camelo-Guarin, 2016; Guerrero-Kommritz & Camelo-Guarin, 2016; Guerrero-Kommritz & Rodriguez-Bermudez, 2018) from the Taganga Bay at Santa Marta, Colombia, revealed three newly described octopus species (i.e., *Octopus tayrona* Guerrero-Kommritz & Camelo-Guarin, 2016; *O. taganga* Guerrero-Kommritz & Camelo-Guarin, 2016, and *Macrotritopus beatrixi* Guerrero-Kommritz & Rodriguez-Bermudez, 2018) and at least seven more remain undescribed (J G-K, pers. obs., 2018), which highlights the currently unrecognized biodiversity of this understudied region. These morphological studies performed in the country are the first step to understand the diversity of Colombian octopod species, and molecular phylogenetic analyses comprise a next step to follow. Therefore, the purpose of this study was to assess the biodiversity of benthic shallow-water octopuses from three locations in the Colombian Caribbean using genetics by constructing the first molecular phylogeny of the family Octopodidae that included Colombian samples. Particular goals were to: (1) provide molecular evidence for recently described species in the area (namely, *O. tayrona*, *O. taganga* and *M. beatrixi*); and (2) contribute to the systematics of the Octopodidae family by adding data from an understudied area of the globe.

## Materials and Methods

### Specimens

**Figure 1:**
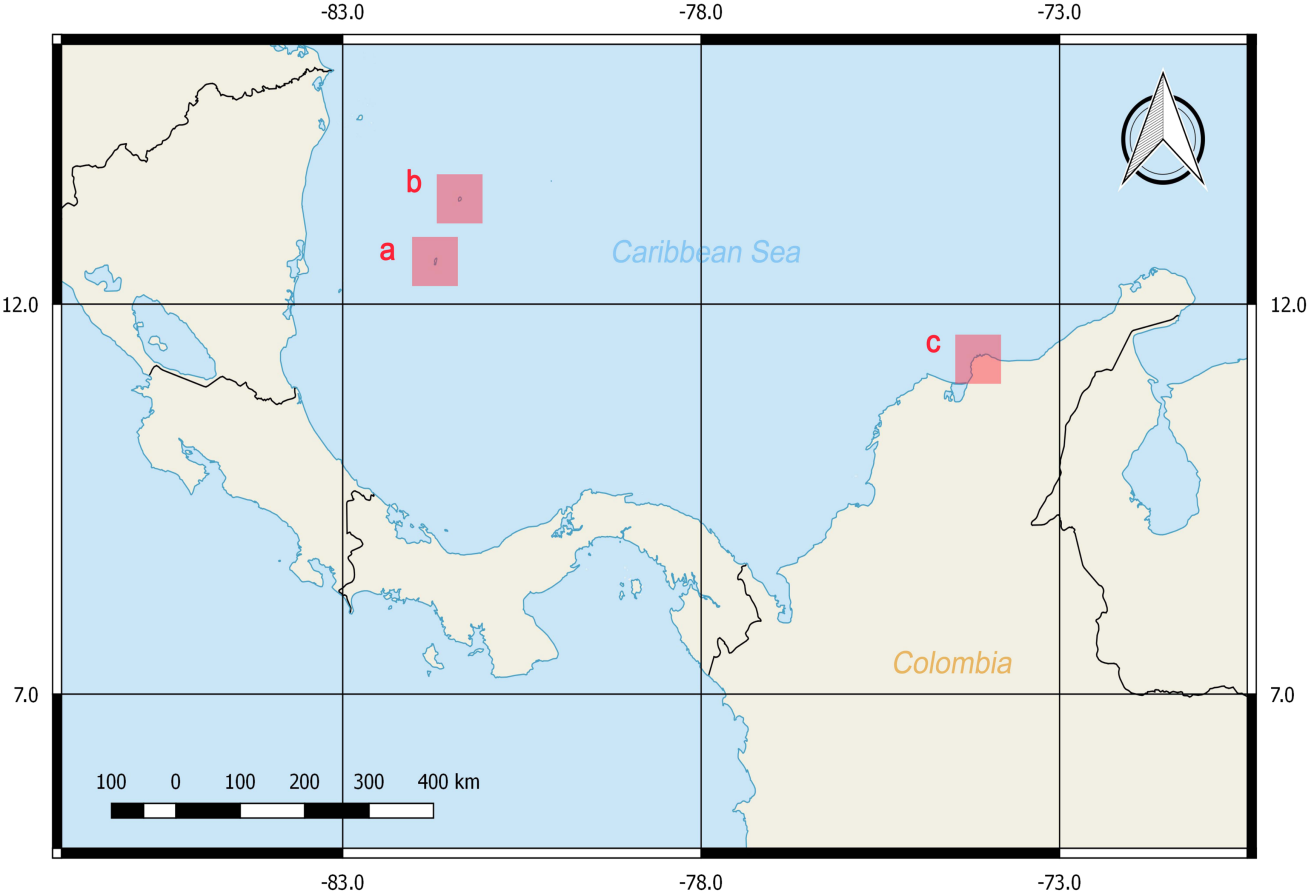
Sampling locations at the Colombian Caribbean Sea. San Andrés Island (A), Old Providence Island (B) and Taganga Bay at Santa Marta (C). Maps were generated with QGIS.

A total of 38 tissue samples and 36 specimens were obtained between February and March of 2016 with the help of local fishermen by hand capture with aid of a hook or a shovel (Guerrero-Kommritz & Camelo-Guarin, 2016; Guerrero-Kommritz & Rodriguez-Bermudez, 2018). The sampling was performed in three areas with depths less than 7 m near the shore at the Caribbean Sea: San Andrés Island, Old Providence Island and Taganga Bay at Santa Marta, Colombia (Fig. 1). Mantle tissues were stored in 90% ethanol at −20 °C until DNA extraction. Voucher specimens were fixed in formalin 4% for 24 to 48 h, preserved in ethanol 70% and identified by morphometries and counts following Guerrero-Kommritz et al. (2016) catalogue for southern Caribbean octopod species. These were deposited in the ANDES Natural History Museum at Los Andes University, Bogota, Colombia (Table S1). The samples corresponded by morphological identifications (summarized in Table S2) to four genera and six described species. Namely, *O. tayrona* (Guerrero-Kommritz & Camelo-Guarin, 2016)*, O. taganga* Guerrero-Kommritz & Camelo-Guarin, 2016, *O. briareus* Robson, 1929*, O. hummelincki* Adam, 1936*, Amphioctopus burryi* Voss, 1950 and *M. beatrixi* (Guerrero-Kommritz & Rodriguez-Bermudez, 2018)*.* Specimens belonging to the *O.* aff. *tayrona* complex (*O.* spB and *O.* spD; Guerrero-Kommritz & Camelo-Guarin, 2016), which are currently being described as new species by J. G-K, and to the genus *Callistoctopus* Taki, 1964 were also found.

Research and collection were approved by the National Environmental Licensing Authority (ANLA, Spanish acronym): Collection Framework Agreement granted to Universidad de los Andes through resolution 1177 of October 9th, 2014 - IBD 0359.

### Molecular techniques

DNA was obtained from tissues by phenol-chloroform extraction and ethanol precipitation following the Coffroth et al. (1992) protocol. Genomic DNA was stored in ultrapure water at −20 °C until polymerase chain reaction (PCR) amplification. The mitochondrial gene *cytochrome c oxidase subunit I* (COI) was amplified using the primers LCO1490 and HC02198 (Folmer et al., 1994) and the nuclear gene *Rhodopsin* (Rho) with the primers 5′-TTGCCTGTACTGTTTGCTAAAGC-3′ and 5′-GCCATCATTTCTTTCATTTGTGCGGCAT -3′(Strugnell, Norman & Drummond, 2004).

Each 15 µl PCR reaction contained the following reagents: 9.2 µl of ddH_2_O, 1.5 µl of 10x buffer, 2.1 µl of 25 mM MgCl_2_, 0.3 µl of 10 mM dNTPs, 0.3 µl of 5 U/ µl *Taq* DNA polymerase (Promega), 0.3 µl of 10 mM of both the forward and reverse primers and 1 µl of 20 ng/ µl DNA. PCR thermal cycling program for COI was: denaturation at 92 °C for 5 min; 35 cycles of 94 °C for 50 s, 54 °C for 1 min and 72 °C for 1 min; and final extension at 72 °C for 7 min completed each PCR. Thermal cycling program for Rho was: denaturation at 94 °C for 2 min; 35 cycles of 94 °C for 40 s, 50 °C for 40 s and 72 °C for 90s; and final extension at 72 °C for 10 min. PCR products were purified with the ExoSAP-IT protocol (Thermo Fisher Scientific) and sequenced by Macrogen Inc (Korea) in both directions using the same primers as for the PCR amplifications. Sequences generated in this study are available under the GenBank accession numbers MG778037–MG778111 (Table S1).

### Taxon sampling

To assess relationships between the individuals found in the three sampling sites and their position within the Octopodidae, the dataset included COI and Rho sequences from different sources (Table S3) of species of the family as the ingroup, and *Enteroctopus dofleini* Wülker, 1910 and *Muusoctopus* Gleadall, 2004 sequences as outgroup. The ingroup was selected taking into account the identification to genus of the specimens collected, which belonged all to the family Octopodidae *sensu* Strugnell et al. (2014). The outgroup species were selected because these were found to be part of the Octopodidae sister group, following the latest Octobrachia phylogeny (Sanchez et al., 2018).

### Phylogenetic analyses and species delimitation

Haplotypes of the COI and Rho datasets were firstly collapsed using the online fasta sequence toolbox FaBox 1.41 (http://www.birc.au.dk/software/fabox). Sequences were aligned with MAFFT v7 (multiple sequence alignment method based on fast Fourier transform (FFT); Katoh & Standley, 2013) with default parameters. Alignments were cleaned with trimAl v1.4 (automated trimming alignment tool; Capella-Gutierrez, Silla-Martinez & Gabaldon, 2009) using a 0.5 gap threshold, 0.25 residue overlap and 90% sequence overlap to erase non-informative sites and sequences. The best-fit model for each dataset was selected with ModelFinder (Kalyaanamoorthy et al., 2017) implemented in IQTree v1.6.2 (Nguyen et al., 2014) using the corrected Akaike Information criterion (AICc). For both COI and Rho datasets, the Generalized Time-Reversible plus Gamma (GTR + G) model of nucleotide substitution was assigned. Best scoring maximum-likelihood trees were constructed with RAxML v8.2.11 (Randomized Axelerated Maximum Likelihood; Stamatakis, 2014) using the rapid bootstrap algorithm, 1,000 bootstrap replicates and 20 alternative runs on distinct starting trees. These resulting trees were finally used as input for the species delimitation analysis with the maximum-likelihood Poisson Tree Process method (PTP; Zhang et al., 2013) as implemented in the web server (https://species.h-its.org/ptp/). Analyses were performed for each gene separately and bootstrap supports >70% were considered significant. Final datasets (sequence files with unique haplotypes), alignments and tree files are available in (Data S1).

## Results

**Figure 2:**
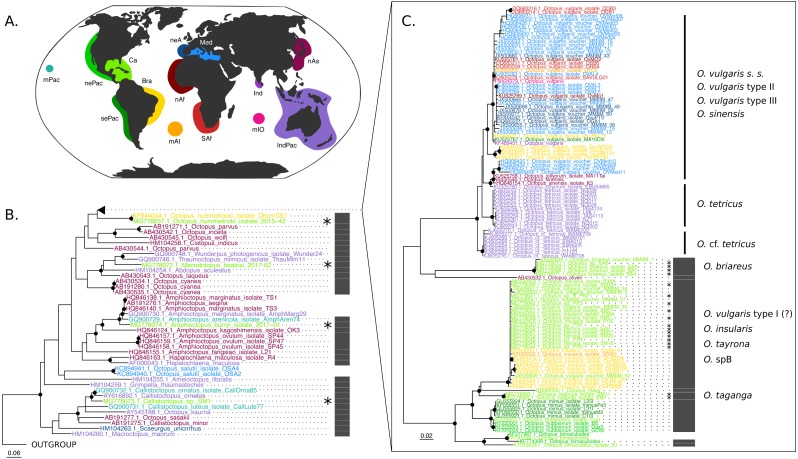
*Cytochrome c oxidase subunit I* (COI) Maximum Likelihood tree. (B–C) Black dots represent bootstrap support over the 70% threshold, and asterisks (*) demark sequences generated in this study. Thick grey bars to the right of the phylogeny represent the delimitation of species using PTP with a bootstrap support higher than 70%. Clades with no such bars were not resolved by the species delimitation analysis. Sequences are colored corresponding to their area of sampling (map in A). Abbreviations as follow - mPac: mid-Pacific; nePac: north-eastern Pacific; sePac: south-eastern Pacific; Ca: Caribbean; Br: Brazil; mAI: mid-Atlantic Islands; SAf: South Africa; nAf: northern Africa; neA: north-eastern Atlantic; Me: Mediterranean Sea; mIO: mid-Indian Ocean Islands; Ind: India; IndPac: Indo-Pacific; nAs: northern Asia. The map template was downloaded from www.onlyGFX.com.

**Figure 3:**
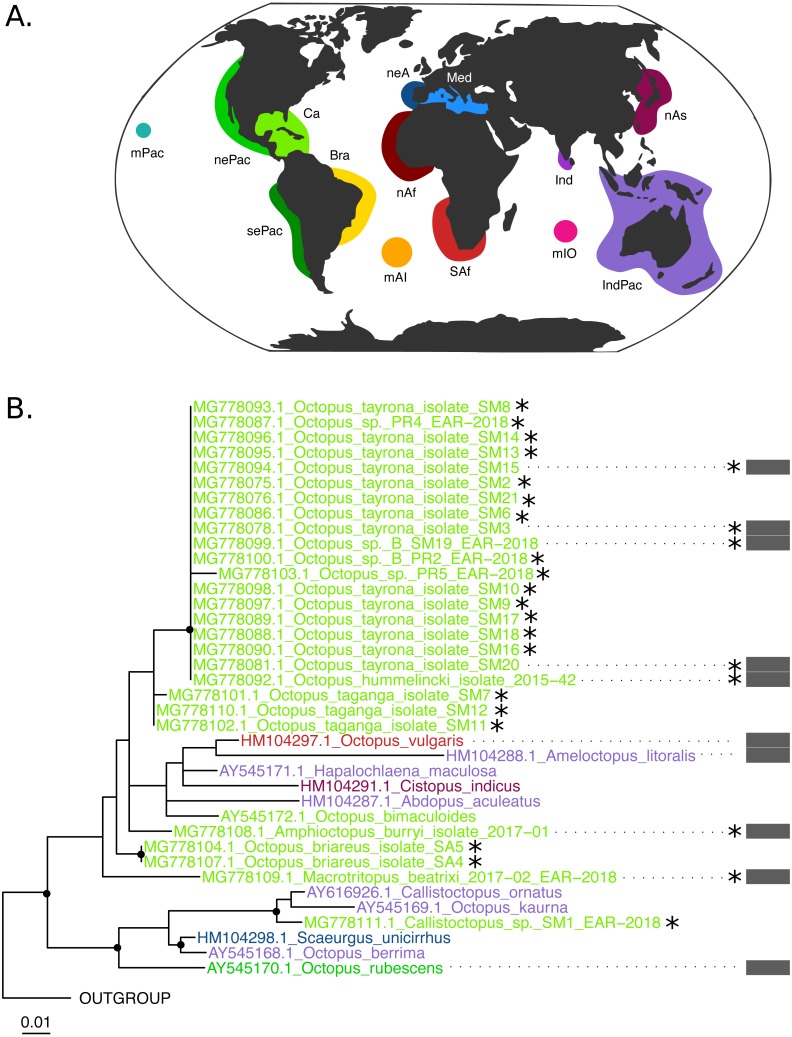
*Rhodopsin* (Rho) Maximum Likelihood tree. (B) Black dots represent bootstrap support over the 70% threshold, and asterisks (*) demark sequences generated in this study. Thick grey bars to the right of the phylogeny represent the delimitation of species using PTP with a bootstrap support higher than 70%. Clades with no such bars were not resolved by the species delimitation analysis. Sequences are colored corresponding to their area of sampling (map in A). Abbreviations as follow - mPac: mid-Pacific; nePac: north-eastern Pacific; sePac: south-eastern Pacific; Ca: Caribbean; Br: Brazil; mAI: mid-Atlantic Islands; SAf: South Africa; nAf: northern Africa; neA: north-eastern Atlantic; Me: Mediterranean Sea; mIO: mid-Indian Ocean Islands; Ind: India; IndPac: Indo-Pacific; nAs: northern Asia. The map template was downloaded from www.onlyGFX.com.

The COI (625 base pairs, 164 sequences) and Rho (235 base pairs, 40 sequences) maximum-likelihood trees of the family Octopodidae are depicted in Figs. 2 and 3, respectively. The COI tree recovered well-supported relationships (bootstrap/BS >70%) mainly at the species level (Fig. 2), whereas deeper branches remain unresolved (BS < 70%). The Rho tree (Fig. 3) included fewer sequences than the COI tree due to the limited availability of this molecular marker in GenBank, resulting in a lower representation of species from the family. Relationships in this analysis were found to be in general discrepant with the COI tree, as detailed below. However, most of these topology inconsistencies were supported below the 70% support level.

The maximum-likelihood approach with the mitochondrial gene (Fig. 2) recovered the monophyly of three species found in the studied area, namely *O. hummelincki, O. briareus* and *O. taganga* (BS = 100%). These were also delimited as single species with the PTP analysis (92%, 77% and 92% BS support, respectively; detailed PTP results in the Data S1). The recently described species *M. beatrixi* resulted as a sister lineage of a well-supported clade (BS = 85%) containing *Wunderpus photogenicus* and *Thaumoctopus mimicus*, although with a low bootstrap support (BS = 65%). The *Callistoctopus* sp. specimen (SM1) was found to be a sister lineage to the two *C. ornatus* representatives from Hawaii and Australia (BS = 85%) and the *A. burryi* sample (2017–01) was found within its genus as a sister lineage to *A. arenicola, A. aegina* and *A. marginatus* (BS = 100%). These three Colombian samples (*i.e., M. beatrixi, Callistoctopus* SM1. and *A. burryi*) were delimited each as single species with PTP (BS = 100%).

Conversely, the monophyly of the species *O. tayrona* could not be resolved. This is because *O. vulgaris* representatives of Puerto Rico and the Lesser Antilles (Caribbean region), and *O. insularis* (Leite et al., 2008) from Brazil and the mid- Atlantic Islands appeared nested in the same clade as *O. tayrona* sequences with a bootstrap support of 100% (Fig. 2). The undescribed species *O.* spD and *O.* spB proposed by Guerrero-Kommritz & Camelo-Guarin (2016) and *O.s* sp. samples from Old Providence Island resulted within this clade as well, in both mitochondrial and nuclear phylogenetic analyses (Figs. 2 and 3). The PTP analysis with COI delimited this clade as one single species (BS = 89%; Fig. 2), whereas the Rho delimitation resulted in the delimitation of individual sequences within this clade as single species (Fig. 3). Moreover, this highly-supported clade resulted in the COI tree (Fig. 2) as sister lineage of *O. maya* (BS = 45%), followed by *O. taganga* (BS = 70%). In the Rho tree the latter resulted to be paraphyletic to the *O.* aff *tayrona* complex (Fig. 3), thus not establishing two separate groups*.* However, this relationship was supported below the 70% bootstrap level (55%). Other species relationships found in the three sampling areas were found to be discordant between the COI and Rho tree. For instance, in the nuclear analysis (Fig. 3) *O. hummelincki* appeared nested within the *O. tayrona* clade (BS = 85%). Conversely, *Callistoctopus* SM1 was found, as in the COI tree, in a clade with *C. ornatus* and *O. kaurna* (BS = 80%; Fig. 3). Moreover, the PTP analysis with the nuclear marker resulted in the delimitation of 10 species supported over the 70% bootstrap level (Fig. 3). However, many of these results were non-concordant with the phylogenetic signal (e.g., delimitation of sequences within the *O.* aff *tayrona/O. taganga* as single species; Fig. 3).

## Discussion

### Regional findings

Our study provides, for the first time, molecular data from octopods found in the Colombian Caribbean and newly described species from this area. The mitochondrial analysis (Fig. 2) resolved the monophyly (BS = 100%) and species delimitation (BS = 92%) of *O. taganga,* the third ocellated species (with *O. hummelincki* and *O. maya*) from the Atlantic*.* Morphologically, *O. taganga* is closely related to *O. hummelincki* and appears to be related to the Pacific Ocean species *Octopus oculifer* (Hoyle, 1904), *Octopus bimaculoides* Pickford and McConnaughey, 1994 and *Octopus bimaculatus* Verrill, 1883 (Guerrero-Kommritz & Camelo-Guarin, 2016). However, preliminary molecular results showed it as more similar to *O. maya* than any other species present in the NCBI database (Guerrero-Kommritz & Camelo-Guarin, 2016). We found in the COI tree (BS = 70%) *O. taganga* as sister lineage of the *O. tayrona/O. insularis* clade and *O. maya* (Fig. 2). This group, together with *O. mimus* and *O. hubbsorum,* showed a close relationship with the ocellated species *O. bimaculoides and O. bimaculatus* (BS = 100%)*.*

The species *O. tayrona* formed a monophyletic group with *O. insularis,* Caribbean *O. vulgaris, Octopus* sp. B, and *Octopus* sp. sequences from Old Providence Island in the COI analysis (Fig. 2, BS = 100%) and was delimited as one single species with PTP (BS = 89%)*.* Until its description, *O. tayrona* and any other large octopus in the Caribbean were identified as *O. vulgaris* (Guerrero-Kommritz & Camelo-Guarin, 2016)*.* However, it can be morphologically distinguished from European *O. vulgaris* by its smaller ligula (*O. tayrona:* 0.6 –1.6 mm; *O. vulgaris:* 1.2 –2.1 mm) and the absence of enlarged suckers in all arms, both in males and females. *O. tayrona* can be additionally distinguished from other similar species, such as *O. insularis,* mainly by its smaller size, the presence of large warts on the arms’ base and the absence of enlarged suckers on arms II and III (Guerrero-Kommritz & Camelo-Guarin, 2016). These features, together with other reproduction-related characters (e.g., relative size of the hectocotylus, presence and form of the calamus) and internal structures related to digestion (e.g., size of liver, teeth on radula, form of salivary glands and structure of stomach), are the base for morphological octopod species delimitation due to their role on ecological adaptation, reproductive isolation and, consequently, speciation. Currently, *O. tayrona* is considered by morphology part of the *O. vulgaris* type I group (Guerrero-Kommritz & Camelo-Guarin, 2016), the species complex form representing the wider Caribbean area (Norman, Finn & Hochberg, 2014). The inclusion of sequences identified as *O. vulgaris* from Puerto Rico and the Lesser Antilles within this clade in our COI tree either supports this claim or, most probably, indicates misidentification of the specimens, as already suggested by Lima et al. (2017). Regardless, these phylogenetic results create a conflict with the morphological species delimitation based on reproduction-related characters of *O. tayrona* and *O. insularis*. Moreover, the Rho tree consistently resulted on a highly supported (BS = 85%) monophyletic group of *O. tayrona, O.* sp. B and *O.* sp. from Old Providence. An *O. insularis* sequence from (González-Gómez et al., 2018) was included in the analysis, but during trimming it was erased due to its low coverage in the alignment. Therefore, together with the lack of rhodopsin sequences for Caribbean *O. vulgaris,* the mitochondrial results regarding *O. tayrona* could not be confirmed with the nuclear marker. Future work using more molecular data would be necessary to follow up these results. This would help to further evaluate the evolutionary relationship between *O. tayrona, O. insularis* and Type I *O. vulgaris* and confirm or reject their taxonomical distinction.

The newly described species *Macrotritopus beatrixi* resulted as an independent lineage in our analyses (Figs. 2 and 3) and was delimited as a single species (COI, BS = 100%; Rho, BS = 93%). So far, it represents the first published sequence of its genus. Thus, data from other *Macrotritopus* species are needed to resolve the genetic distinction of this new species. Moreover, our COI phylogeny and PTP results suggest that *Callistoctopus* SM1 from Santa Marta represents one independent lineage and a single species (BS = 100%), different from all other representatives of this genus included in the analysis (Fig. 2). A new species of *Callistoctopus* is currently under description by J. G-K., expected to be closely related to *C. macropus* Risso, 1826 from the east Atlantic Ocean and Mediterranean Sea (due to morphological similarities; J G-K, pers. obs., 2018). *Callistoctopus macropus* is also reported for the southern Caribbean (Díaz, Ardila & Gracia, 2000; Guerrero-Kommritz & Camelo-Guarin, 2016). However, after analyzing specimens from France the morphometrics and counts were different, and we considered this a misidentification (research in process J. G-K). Unfortunately, the sequence generated here was taken from an individual whose whole body could not be collected. Therefore, its morphological identification to species level was not possible to achieve. Moreover, due to the lack of sequences of *C. macropus* in databases, their evolutionary relationship could not be molecularly assessed here and warrants further investigation.

### Contributions to the Octopodidae systematics

Our COI and Rho phylogenies help to fulfill the taxonomic gap in the Octopodidae family of species previously lacking molecular information and thus not considered in the phylogenetic systematics of the family (i.e*., O. tayrona, O taganga, A. burryi* and *M. beatrixi*). For instance, the *Macrotritopus beatrixi* sample contributes the first sequences of its genus, providing new insights on the Octopodidae genus-level systematics. Additionally, the inclusion of *O. tayrona* in our COI analysis*,* considered morphologically as part of the Type I *O. vulgaris* group*,* allowed the inference of the evolutionary relationship of this species with other forms of the species complex. Our results suggest that *O. tayrona* is more closely related to other species also found in the New World (e.g*., O. taganga* from Colombia*, O. hubbsorum, O. bimaculatus* and *O. maya* from Mexico, *O. mimus* from Chile and Peru, *O. bimaculoides* from California, USA) than to the *O. vulgaris* forms from the Old World and Brazil (Fig. 2). Moreover, its genetic distinction with *O. insularis* remains unresolved.

Our mitochondrial analysis showed a better-resolved topology, with less polytomies and more significantly-supported branches (BS > 70%) than the Rho tree. This could be explained by the general smaller effective population size of mitochondrial DNA compared to nuclear genes (Moore, 1995). This leads to a higher probability of coalescence in short internodes and can result on a more accurate recovery of the species-tree topology. However, even a well-resolved gene tree can hardly resemble a species tree due to incomplete lineage sorting and DNA barcodes are known to decrease their performance on poorly sampled groups (Meyer & Paulay, 2005). Moreover, recent genome-wide findings on the *O. vulgaris* species complex (Amor et al., 2019) highlight that phylogenetic analyses using only mitochondrial DNA underestimate octopus species diversity. Thus, despite the better performance of our COI analysis, more informative nuclear markers are needed to complement our results.

The number of cephalopod species in the Caribbean should increase as more surveys are made (Judkins et al., 2010; Miloslavich et al., 2010). Here we show a first attempt to fulfill taxonomic gaps on the octopod species of an understudied area of the highly diverse Caribbean. However, more sampling and taxonomic efforts are still needed to contribute to the local and the world-wide picture of the Octopodidae systematics. Therefore, for future studies we suggest the inclusion of more taxa poorly represented in databases to account for incomplete taxon sampling and further tests using more molecular information to account for insufficient single-locus resolution.

## Conclusions

Here we present the first molecular approach to the octopus fauna from the southern Caribbean in Colombia. Our mitochondrial analysis supports the monophyly of species found in the Colombian Caribbean, like *Octopus taganga*, *O. hummelincki* and *O. briareus*. The genetic distinction of the species *O. insularis* and *O. tayrona* was not resolved, as these were found in one unresolved clade and delimited as one species together with Caribbean *Octopus vulgaris* and *Octopus* aff*. tayrona* species (*O.* spB). Moreover, our results suggest a distant relationship of the *O. vulgaris*-like species from the Caribbean region (*e.g., O. tayrona* and *O. insularis*) from the *O. vulgaris* species complex forms of the Old World and Brazil. Lastly, the newly described species *Macrotritopus beatrixi* was delimited as a single species but its relationship to other species of its genus remains unknown. Altogether, our work contributes to the fulfilment of taxonomic gaps in the Octopodidae family and adds molecular evidence for recent descriptions in this area. We suggest for future studies to use more molecular markers to compensate for insufficient single-locus resolution. Furthermore, molecular data of poorly represented species should be amended to databases to improve taxon sampling.

##  Supplemental Information

10.7717/peerj.7300/supp-1 Supplemental Information 1 Tables S1, S2, S3 Table S1: GenBank accession numbers and ANDES-E codes (ANDES Natural History Museum, Los Andes University, Bogota, Colombia. Samples without museum code are in the process of entering the collection; NA (not applicable) is designated for samples that lack specimen for voucher.
Table S2: Summary of morphological characters used for the identification of octopus species from the southern Caribbean found in this study.
Table S3: Accession numbers, sampling location, reference and area (as defined in Fig. 2 and 3) of sequences used in the phylogenetic analyses (after collapsing haplotypes and cleaning alignments). All sequences were downloaded from GenBank.

10.7717/peerj.7300/supp-2 Supplemental Information 2 Sequences and alignments used in this study, and resulting COI and Rho phylogenetic trees Raw alignments correspond to untrimmed alignments (output of MAFFT); clean alignments correspond to trimmed alignments using trimAl.

## Additional Information and Declarations

### DNA Deposition

The following information was supplied regarding the deposition of DNA sequences: The sequences generated here are accesible via GenBank accession numbers MG778037 to MG778111. Sequences can be also found in Data S1.

### Data Availability

The following information was supplied regarding data availability: Sequences and alignments used for the phylogenetic analyses and resulting trees for both *cytochrome c oxidase subunit I* and rhodopsin genes are available in Data S1.
