# Supplementary material for: First molecular approach to the octopus fauna from the southern Caribbean
Source: PeerJ. 2019 Jul 29;7:e7300. doi: 10.7717/peerj.7300 (PMC6673601; doi:10.7717/peerj.7300)
Supplement: Supplemental Information 1 — Table S1: GenBank accession numbers and ANDES-E codes (ANDES Natural History Museum, Los Andes University, Bogota, Colombia. Samples without museum code are in the process of entering the collection; NA (not applicable) is designated for samples that lack specimen for voucher. Table S2: Summary of morphological characters used for the identification of octopus species from the southern Caribbean found in this study. Table S3: Accession numbers, sampling location, reference and area (as defined in Fig. 2 and 3) of sequences used in the phylogenetic analyses (after collapsing haplotypes and cleaning alignments). All sequences were downloaded from GenBank. [file peerj-07-7300-s001.docx]

**First molecular approach to the octopus’ fauna from the southern Caribbean**

Marine Biodiversity

Elena A. Ritschard^1,2^, Jürgen Guerrero-Kommritz^3^, Juan A. Sanchez^1^

^1^Laboratorio de Biología Molecular Marina (BIOMMAR), Departamento de Ciencias Biológicas-Facultad de Ciencias, Universidad de Los Andes, Carrera 1E No. 18A – 10, Bogotá, Colombia

^2^Department of Molecular Evolution and Development, University of Vienna, Althanstrasse 14 (UZA1), Vienna, Austria

^3^Fundación Fundabas, Bogotá, Colombia

Correspondence author:

Elena A. Ritschard

[ea.ritschard10@uniandes.edu.co](mailto:ea.ritschard10@uniandes.edu.co)

**Table S1. GenBank accession numbers and ANDES-E codes (ANDES Natural History Museum, Los Andes University, Bogota, Colombia) of the samples obtained in the three sampling locations.** Samples without museum code are in the process of entering the collection; NA (not applicable) is designated for samples that lack specimen for voucher.

| **Sample code** | **Species** | **Museum code (ANDES- E)** | **Genbank accession number** | | **Sampling location** |
| --- | --- | --- | --- | --- | --- |
|  |  |  | **COI** | **Rho** |  |
| PR1 | *Octopus* sp | - | MG778040 | MG778085 | Old Providence |
| PR2 | *Octopus* spB | - | MG778041 | MG778100 | Old Providence |
| PR3 | *Octopus* sp | - | MG778042 | MG778077 | Old Providence |
| PR4 | *Octopus* sp | - | MG778043 | MG778087 | Old Providence |
| PR5 | *Octopus* sp | - | MG778044 | MG778103 | Old Providence |
| PR6 | *Octopus* sp | - | MG778045 | MG778084 | Old Providence |
| SA1 | *Octopus briareus* | 4955 | MG778066 | MG778105 | San Andrés |
| SA2 | *Octopus briareus* | 4953 | MG778067 | - | San Andrés |
| SA3 | *Octopus briareus* | 4957 | MG778068 | MG778106 | San Andrés |
| SA4 | *Octopus briareus* | 4956 | MG778071 | MG778107 | San Andrés |
| SA5 | *Octopus briareus* | 4954 | MG778069 | MG778104 | San Andrés |
| SA6 | *Octopus* sp | 4958 | MG778070 | MG778091 | San Andrés |
| SA7 | *Octopus* sp | 4959 | MG778046 | MG778083 | San Andrés |
| SA8 | *Octopus tayrona* | 4952 | MG778055 | MG778082 | San Andrés |
| SM1 | *Callistoctopus* sp | NA | MG778073 | MG778111 | Santa Marta |
| SM2 | *Octopus tayrona* | 4979 | MG778038 | MG778075 | Santa Marta |
| SM3 | *Octopus tayrona* | 4978 | MG778053 | MG778078 | Santa Marta |
| SM4 | *Octopus tayrona* | 4977 | MG778052 | MG778079 | Santa Marta |
| SM5 | *Octopus* spD | 4976 | MG778058 | MG778080 | Santa Marta |
| SM6 | *Octopus tayrona* | 4972 | MG778059 | MG778086 | Santa Marta |
| SM7 | *Octopus taganga* | 4973 | MG778063 | MG778101 | Santa Marta |
| SM8 | *Octopus tayrona* | 4971 | MG778057 | MG778093 | Santa Marta |
| SM9 | *Octopus tayrona* | 4970 | MG778054 | MG778097 | Santa Marta |
| SM10 | *Octopus tayrona* | 4974 | MG778060 | MG778098 | Santa Marta |
| SM11 | *Octopus taganga* | 4975 | MG778065 | MG778102 | Santa Marta |
| SM12 | *Octopus taganga* | 4961 | MG778064 | MG778110 | Santa Marta |
| SM13 | *Octopus tayrona* | 4963 | MG778062 | MG778095 | Santa Marta |
| SM14 | *Octopus tayrona* | 4962 | MG778061 | MG778096 | Santa Marta |
| SM15 | *Octopus tayrona* | 4960 | MG778039 | MG778094 | Santa Marta |
| SM16 | *Octopus tayrona* | 4966 | MG778056 | MG778090 | Santa Marta |
| SM17 | *Octopus tayrona* | 4967 | MG778047 | MG778089 | Santa Marta |
| SM18 | *Octopus tayrona* | 4968 | MG778048 | MG778088 | Santa Marta |
| SM19 | *Octopus* spB | 4965 | MG778049 | MG778099 | Santa Marta |
| SM20 | *Octopus tayrona* | 4964 | MG778050 | MG778081 | Santa Marta |
| SM21 | *Octopus tayrona* | 4969 | MG778051 | MG778076 | Santa Marta |
| 2017-01 | *Amphioctopus* *burryi* | - | MG778074 | MG778108 | Santa Marta |
| 2017-02 | *Macrotritopus beatrixi* | - | MG778072 | MG778109 | Santa Marta |
| 2015-42 | *Octopus hummelincki* | - | MG778037 | MG778092 | Santa Marta |

**Table S2. Summary of morphological characters used for the identification of octopus species from the southern Caribbean found in this study.** ML (Mantle Length) refers to the maximum size known for each species. Ocellus refers to a dark spot present between the base of arms II and III. Mantle-arm ratio is the proportion of the ML to the longest arm. Enlarged suckers are specialized suckers for mating purposes found on these species in arms II and III. Funnel organ refers to the shape of a gland at the inner surface of the funnel (W-shaped or V V-shaped). SC HAL is the sucker count on the hectocotylized arm (HA). Calamus index is the percentage of the calamus length to the ligula length (calamus length/ligula length x 100). Ligula index is the percentage of the ligula length to the HA length (ligula length/HAL x 100). The identification to species by morphometrics of the *Callistoctopus* sample SM1 could not be assessed, but we include here the specifications for the species currently under description by J. G-K (*).

| **Species** | **ML (mm)** | **Ocellus** | **Mantle-arm ratio** | **Longest arm pair** | **Enlarged suckers** | **Lamellas in demi-**  **branch** | **Funnel organ** | **SC HA** | **Calamus index** | **Ligula index** |
| --- | --- | --- | --- | --- | --- | --- | --- | --- | --- | --- |
| *Octopus taganga* | 81 | Yes | 2 - 3 | III | No | 7 - 10 | W | 80 | 38 - 50 | 0.8 – 1.8 |
| *Octopus hummelincki* | 50l | Yes | 2 – 2.5 | II | Yes | 6 - 7 | W | 80 | 52-56 | 2-6 |
| *Octopus* *tayrona* | 127 | No | 2 - 3 | II =III | No | 9 - 12 | W | 112 - 135 | 20 - 50 | 0.4 – 1.6 |
| *Octopus* spB | 76 | No | 3 - 4 | II = IV | No | 11 | V V | 100 – 108 | 40 – 50 | 1.5 – 1.9 |
| *Octopus* spD | 86 | No | 3 - 4 | II | No | 10-11 | W | 152 – 156 | 0 | 0.65 – 0.88 |
| *Octopus briareus* | 150 | No | 5 - 7 | II = III | No | 6 - 7 | W | 141 - 182 | 17,5 | 3 |
| *Amphioctopus burryi* | 50l | No | 3 | IV | No | 10 | W | 90 | 40 | 1.6 |
| *Callistoctopus* spA* | 75 | No | 6 - 7 | I | No | 12 | W | 152 | 14.3 | 4.8 |
| *Macrotritopus* *beatrixi* | 70 | No | 5 - 8 | III | No | 10 - 11 | W | 98 | 25 | 1-1.25 |

**Table S3. Accession numbers, sampling location, reference and area (as defined in Fig. 2 and 3) of sequences used in the phylogenetic analyses (after collapsing haplotypes and cleaning alignments).** All sequences were downloaded from GenBank. OG: outgroup; mPac: mid-Pacific; nePac: north-eastern Pacific; sePac: south-eastern Pacific; Ca: Caribbean; Br: Brazil; mAI: mid-Atlantic Islands; SAf: South Africa; nAf: northern Africa; neA: north-eastern Atlantic; Me: Mediterranean Sea; mIO: mid-Indian Ocean Islands; Ind: India; IndPac: Indo-Pacific; nAs: northern Asia.

| **Species** | **COI** | **Rho** | **Sampling Place** | **Reference** | **Area** |
| --- | --- | --- | --- | --- | --- |
| *Enteroctopus dofleini* | GU802397 | - | British Columbia, Queen Charlotte Sound, Canada |  | OG |
| *Enteroctopus dofleini* | - | AY545174 | - | Strugnell et al., 2004 | OG |
| *Muusoctopus sp* | GU073624 | - | - | Undheim et al., 2010 | OG |
| *Muusoctopus sp* | - | AY545173 | - | Strugnell et al., 2004 | OG |
| *Abdopus aculeatus* | H M104254 | HM104287 | Orpheus Island, Australia | Strugnell et al., 2014 | IndPac |
| *Ameloctopus litoralis* | HM104255 | HM104288 | Dudley Point, N.T., Australia | Strugnell et al., 2014 | IndPac |
| *Amphioctopus aegina* | AB191276 | - | Irino, Kochi, Japan | Takumiya et al., 2005 | nAs |
| *Amphioctopus arenicola* | GQ900729 | - | Hawaii, USA | Huffard et al., 2010 | mPac |
| *Amphioctopus fangsiao* | HQ846155 | - | Xiamen, China | Dai et al., 2012 | nAs |
| *Amphioctopus kagoshimensis* | HQ846124 | - | Xiamen, China | Dai et al., 2012 | nAs |
| *Amphioctopus marginatus* | GQ900730 | - | Indonesia | Huffard et al., 2010 | IndPac |
| *Amphioctopus marginatus* | HQ846138 | - | Xiamen, China | Dai et al., 2012 | nAs |
| *Amphioctopus marginatus* | HQ846140 | - | Xiamen, China | Dai et al., 2012 | nAs |
| *Amphioctopus ovulum* | HQ846157 | - | Xiamen, China | Dai et al., 2012 | nAs |
| *Amphioctopus ovulum* | HQ846158 | - | Xiamen, China | Dai et al., 2012 | nAs |
| **Species** | **COI** | **Rho** | **Sampling Place** | **Reference** | **Area** |
| *Amphioctopus ovulum* | HQ846159 | - | Xiamen, China | Dai et al., 2012 | nAs |
| *Callistoctopus luteus* | GQ900731 | - | Hawaii, USA | Huffard et al., 2010 | mPac |
| *Callistoctopus minor* | AB191275 | - | Akashi, Osaka, Japan | Takumiya et al., 2005 | nAs |
| *Callistoctopus ornatus* | AY616892 | AY616926 | Australia | Strugnell et al., 2005 | IndPac |
| *Callistoctopus ornatus* | GQ900732 | - | Hawaii, USA | Huffard et al., 2010 | mPac |
| *Cistopus indicus* | HM104258 | HM104291 | Taichung Fish Market, Taiwan | Strugnell et al., 2014 | nAs |
| *Grimpella thaumastocheir* | HM104259 | - | Pt. Victoria Jetty, S.A., Australia | Strugnell et al., 2014 | IndPac |
| *Hapalochlaena maculosa* | AF000043 | - | Victoria, Australia | Carlini et al., 1999 | IndPac |
| *Hapalochlaena maculosa* | HQ846163 | - | Lingao, China | Dai et al., 2012 | nAs |
| *Hapalochlaena maculosa* | - | AY545171 | Victoria, Australia | Strugnell et al., 2004 | IndPac |
| *Macroctopus maorum* | HM104260 | - | Victoria, Australia | Strugnell et al., 2014 | IndPac |
| *Octopus berrima* | - | AY545168 | Venus Bay, Australia | Strugnell et al., 2004 | IndPac |
| *Octopus bimaculatus* | KT335828 | - | Mexico | Pliego-Cardenas et al., 2016 | Ca |
| *Octopus bimaculoides* | AF377967 | - | California, USA | Carlini et al., 2001 | nePac |
| *Octopus bimaculoides* | AY545189 | AY545172 | Galveston, TX, USA | Strugnell et al., 2004 | Ca |
| *Octopus bimaculoides* | KF774309 | - | Gulf of California, Mexico |  | nePac |
| *Octopus briareus* | JX500618 | - | Curazao |  | Ca |
| *Octopus cf tetricus* | KJ605262 | - | Woodmans Point, Australia | Amor et al., 2014 | IndPac |
| *Octopus cf tetricus* | KJ605263 | - | Town Jetty, Australia | Amor et al., 2014 | IndPac |
| *Octopus cf tetricus* | KJ605264 | - | Cape Le Grand, Western Australia | Amor et al., 2014 | IndPac |
| *Octopus cf tetricus* | KJ605268 | - | Esperance, Australia | Amor et al., 2014 | IndPac |
| **Species** | **COI** | **Rho** | **Sampling Place** | **Reference** | **Area** |
| *Octopus cf tetricus* | KJ605269 | - | Mandurah, Australia | Amor et al., 2014 | IndPac |
| *Octopus cf tetricus* | KJ605270 | - | Mandurah, Australia | Amor et al., 2014 | IndPac |
| *Octopus cf tetricus* | KJ605276 | - | Mandurah, Western Australia | Amor et al., 2014 | IndPac |
| *Octopus cf tetricus* | KJ605277 | - | Mandurah, Australia | Amor et al., 2014 | IndPac |
| *Octopus cyanea* | AB191280 | - | Nago, Okinawa, Japan | Takumiya et al., 2005 | nAs |
| *Octopus cyanea* | AB430534 | - | Tokyo, Ogasawara Island, Japan | Kaneko et al., 2011 | nAs |
| *Octopus cyanea* | AB430535 | - | Okinawa, Nakagusuku, Japan | Kaneko et al., 2011 | nAs |
| *Octopus hubbsorum* | KF225001 | - | Pacific Coast of Mexico | Pliego-Cardenas et al., 2014 | nePac |
| *Octopus hubbsorum* | KF225003 | - | Pacific Coast of Mexico | Pliego-Cardenas et al., 2014 | nePac |
| *Octopus hubbsorum* | KF225004 | - | Pacific Coast of Mexico | Pliego-Cardenas et al., 2014 | nePac |
| *Octopus hubbsorum* | KF225005 | - | Colombia | Pliego-Cardenas et al., 2014 | Ca |
| *Octopus hummelincki* | KF844044 | - | Ceara, Brazil | De Luna Sales et al., 2013 | Bra |
| *Octopus incella* | AB430542 | - | Japan | Kaneko et al., 2011 | nAs |
| *Octopus insularis* | KF844000 | - | Bragana, Brazil | De Luna Sales et al., 2013 | Bra |
| *Octopus insularis* | KF844003 | - | Fortaleza, Brazil | De Luna Sales et al., 2013 | Bra |
| *Octopus insularis* | KF844010 | - | Natal, Brazil | De Luna Sales et al., 2013 | Bra |
| *Octopus insularis* | KF844016 | - | Natal, Brazil | De Luna Sales et al., 2013 | Bra |
| *Octopus insularis* | KF844018 | - | Baia da Traicao, Brazil | De Luna Sales et al., 2013 | Bra |
| *Octopus insularis* | KF844020 | - | Recife, Brazil | De Luna Sales et al., 2013 | Bra |
| *Octopus insularis* | KF844022 | - | Barra Grande, Brazil | De Luna Sales et al., 2013 | Bra |
| *Octopus insularis* | KF844024 | - | Barra Grande, Brazil | De Luna Sales et al., 2013 | Bra |
| *Octopus insularis* | KF844025 | - | Salvador, Brazil | De Luna Sales et al., 2013 | Bra |
| **Species** | **COI** | **Rho** | **Sampling Place** | **Reference** | **Area** |
| *Octopus insularis* | KP056552 | - | Lemon Valley, Saint Helena | Amor et al., 2015 | mAI |
| *Octopus insularis* | MH550422 | - | Gulf of Mexico | Gonzalez-Gomez et al., 2018 | Ca |
| *Octopus insularis* | MH550423 | - | Gulf of Mexico | Gonzalez-Gomez et al., 2018 | Ca |
| *Octopus insularis* | MH550425 | - | Gulf of Mexico | Gonzalez-Gomez et al., 2018 | Ca |
| *Octopus jollyorum* | KU525758 | - | Yilan County, Dasi, Taiwan | Amor et al., 2016 | nAs |
| *Octopus jollyorum* | KU525760 | - | Kermadecs, North Meyer Island, New Zealand | Amor et al., 2016 | IndPac |
| *Octopus kaurna* | AY545188 | AY545169 | Edithburgh Jetty, South Australia | Strugnell et al., 2004 | IndPac |
| *Octopus laqueus* | AB430543 | - | Japan | Kaneko et al., 2011 | nAs |
| *Octopus maya* | GU362545 | - | Yucatan, Mexico | Juarez et al., 2012 | Ca |
| *Octopus mimus* | GU355923 | - | Coloso, Chile | Acosta-Jofre et al., 2012 | sePac |
| *Octopus mimus* | GU355924 | - | Callao, Peru | Acosta-Jofre et al., 2012 | sePac |
| *Octopus mimus* | GU355925 | - | Callao, Peru | Acosta-Jofre et al., 2012 | sePac |
| *Octopus mimus* | KP056550 | - | Chile | Amor et al., 2015 | sePac |
| *Octopus mimus* | KP056551 | - | Chile | Amor et al., 2015 | sePac |
| *Octopus oliveri* | AB430532 | - | Japan | Kaneko et al., 2011 | nAs |
| *Octopus parvus* | AB191271 | - | Shirahama, Wakayama, Japan | Takumiya et al., 2005 | nAs |
| *Octopus parvus* | AB430544 | - | Japan | Kaneko et al., 2011 | nAs |
| *Octopus rubescens* | - | AY545170 | North Vancouver, BC, Canada | Strugnell et al., 2004 | nePac |
| *Octopus salutii* | KC894940 | - | Mediterranean Sea |  | Med |
| *Octopus salutii* | KC894941 | - | Mediterranean Sea |  | Med |
| *Octopus sasakii* | AB191277 | - | Tokushima, Tokushima, Japan | Takumiya et al., 2005 | nAs |
| **Species** | **COI** | **Rho** | **Sampling Place** | **Reference** | **Area** |
| *Octopus sinensis* | AB430546 | - | Hyougo, Akashi, Futami, Japan | Kaneko et al., 2011 | nAs |
| *Octopus sinensis* | HQ846154 | - | Xiamen, China | Dai et al., 2012 | nAs |
| *Octopus tetricus* | KJ605247 | - | Wallaga Lake, Australia | Amor et al., 2014 | IndPac |
| *Octopus tetricus* | KJ605248 | - | Wallaga Lake, Australia | Amor et al., 2014 | IndPac |
| *Octopus tetricus* | KJ605249 | - | Wallaga Lake, New South Wales, Australia | Amor et al., 2014 | IndPac |
| *Octopus tetricus* | KJ605250 | - | Wallaga Lake, Australia | Amor et al., 2014 | IndPac |
| *Octopus tetricus* | KJ605251 | - | Narooma, New South Wales, Australia | Amor et al., 2014 | IndPac |
| *Octopus tetricus* | KJ605252 | - | Shoal Bay, New South Wales, Australia | Amor et al., 2014 | IndPac |
| *Octopus tetricus* | KJ605253 | - | Port Stephens, Australia | Amor et al., 2014 | IndPac |
| *Octopus tetricus* | KJ605254 | - | Flinders Island, Tasmania, Australia | Amor et al., 2014 | IndPac |
| *Octopus tetricus* | KJ605256 | - | Flinders Island, Australia | Amor et al., 2014 | IndPac |
| *Octopus tetricus* | KJ605258 | - | Leigh, New Zealand | Amor et al., 2014 | IndPac |
| *Octopus tetricus* | KJ605261 | - | Leigh, New Zealand | Amor et al., 2014 | IndPac |
| *Octopus vulgaris* | DQ683205 | - | Tristan da Cunha | Teske et al., 2007 | mAI |
| *Octopus vulgaris* | DQ683209 | - | Hout Bay, South Africa | Teske et al., 2007 | SAf |
| *Octopus vulgaris* | DQ683211 | - | Struisbaai, South Africa | Teske et al., 2007 | SAf |
| *Octopus vulgaris* | DQ683214 | - | Durban, South Africa | Teske et al., 2007 | SAf |
| *Octopus vulgaris* | DQ683216 | - | Durban, South Africa | Teske et al., 2007 | SAf |
| *Octopus vulgaris* | DQ683221 | - | Galicia, Spain | Teske et al., 2007 | neA |
| *Octopus vulgaris* | FN424379 | - | St. Paul and Amsterdam Islands | Guerra et al., 2010 | mIO |
| *Octopus vulgaris* | HQ908426 | - | Turkey (Mediterranean Sea) | Keskin & Atar, 2011 | Med |
| *Octopus vulgaris* | HQ908427 | - | Turkey (Mediterranean Sea) | Keskin & Atar, 2011 | Med |
| **Species** | **COI** | **Rho** | **Sampling Place** | **Reference** | **Area** |
| *Octopus vulgaris* | HQ908428 | - | Turkey (Mediterranean Sea) | Keskin & Atar, 2011 | Med |
| *Octopus vulgaris* | HQ908429 | - | Turkey (Mediterranean Sea) | Keskin & Atar, 2011 | Med |
| *Octopus vulgaris* | HQ908430 | - | Turkey (Mediterranean Sea) | Keskin & Atar, 2011 | Med |
| *Octopus vulgaris* | HQ908431 | - | Turkey (Mediterranean Sea) | Keskin & Atar, 2011 | Med |
| *Octopus vulgaris* | HQ908432 | - | Turkey (Mediterranean Sea) | Keskin & Atar, 2011 | Med |
| *Octopus vulgaris* | HQ908433 | - | Turkey (Mediterranean Sea) | Keskin & Atar, 2011 | Med |
| *Octopus vulgaris* | HQ908434 | - | Turkey (Mediterranean Sea) | Keskin & Atar, 2011 | Med |
| *Octopus vulgaris* | HQ908435 | - | Turkey (Mediterranean Sea) | Keskin & Atar, 2011 | Med |
| *Octopus vulgaris* | HQ908436 | - | Turkey (Mediterranean Sea) | Keskin & Atar, 2011 | Med |
| *Octopus vulgaris* | JX500622 | - | Dominica, Lesser Antilles |  | Ca |
| *Octopus vulgaris* | JX500626 | - | France |  | Med |
| *Octopus vulgaris* | JX500629 | - | Ikaria, Greece |  | Med |
| *Octopus vulgaris* | JX500630 | - | Samos, Greece |  | Med |
| *Octopus vulgaris* | JX500631 | - | Samos, Greece |  | Med |
| *Octopus vulgaris* | JX500632 | - | Samos, Greece |  | Med |
| *Octopus vulgaris* | JX500633 | - | Samos, Greece |  | Med |
| *Octopus vulgaris* | JX500636 | - | Samos, Greece |  | Med |
| *Octopus vulgaris* | JX500649 | - | Puerto Rico |  | Ca |
| *Octopus vulgaris* | JX500651 | - | Puerto Rico |  | Ca |
| *Octopus vulgaris* | JX500652 | - | Puerto Rico |  | Ca |
| *Octopus vulgaris* | JX500654 | - | Puerto Rico |  | Ca |
| *Octopus vulgaris* | JX500655 | - | Spain |  | neA |
| **Species** | **COI** | **Rho** | **Sampling Place** | **Reference** | **Area** |
| *Octopus vulgaris* | JX500660 | - | Spain |  | neA |
| *Octopus vulgaris* | JX500664 | - | Spain |  | neA |
| *Octopus vulgaris* | JX500666 | - | Spain |  | neA |
| *Octopus vulgaris* | JX500676 | - | Spain |  | neA |
| *Octopus vulgaris* | JX500677 | - | Spain |  | neA |
| *Octopus vulgaris* | KF489451 | - | Kerala, Neendakara, India |  | Ind |
| *Octopus vulgaris* | KF844026 | - | Amapa, Brazil | De Luna Sales et al., 2013 | Bra |
| *Octopus vulgaris* | KF844027 | - | Para, Brazil | De Luna Sales et al., 2013 | Bra |
| *Octopus vulgaris* | KF844030 | - | Para, Brazil | De Luna Sales et al., 2013 | Bra |
| *Octopus vulgaris* | KF844031 | - | Salvador, Brazil | De Luna Sales et al., 2013 | Bra |
| *Octopus vulgaris* | KF844040 | - | Santa Catarina, Brazil | De Luna Sales et al., 2013 | Bra |
| *Octopus vulgaris* | KF844042 | - | Portugal | De Luna Sales et al., 2013 | neA |
| *Octopus vulgaris* | KJ605279 | - | Port Elizabeth, South Africa | Amor et al., 2014 | SAf |
| *Octopus vulgaris* | KJ605280 | - | Perpignan, France | Amor et al., 2014 | Med |
| *Octopus vulgaris* | KJ605282 | - | Perpignan, France | Amor et al., 2014 | Med |
| *Octopus vulgaris* | KJ605283 | - | Perpignan, France | Amor et al., 2014 | Med |
| *Octopus vulgaris* | KJ605284 | - | Perpignan, France | Amor et al., 2014 | Med |
| *Octopus vulgaris* | KU525761 | - | Cabo Blanco, Morocco | Amor et al., 2016 | nAf |
| *Octopus vulgaris* | KU525767 | - | Juan Fernandez Island, Chile | Amor et al., 2016 | sePac |
| *Octopus vulgaris* | KU525769 | - | Cabo Blanco, Morocco | Amor et al., 2016 | nAf |
| *Octopus vulgaris* | - | HM104297 | Port Elizabeth, South Africa | Strugnell et al., 2014 | SAf |
| **Species** | **COI** | **Rho** | **Sampling Place** | **Reference** | **Area** |
| *Octopus wolfi* | AB430545 | - | Japan | Kaneko et al., 2011 | nAs |
| *Scaeurgus unichirrhus* | HM104263 | HM104298 | North Eastern Atlantic | Strugnell et al., 2014 | neA |
| *Thaumoctopus mimicus* | GQ900746 | - | Sulawesi, Indonesia | Huffard et al., 2010 | IndPac |
| *Wunderpus photogenicus* | GQ900748 | - | Sulawesi, Indonesia | Huffard et al., 2010 | IndPac |

**References**

Acosta-Jofré MS, Sahade R, Laudien J, Chiappero MB (2012) A contribution to the understanding of phylogenetic relationships among species of the genus Octopus ( Octopodidae : Cephalopoda ). Sci Mar 76:311–318 . doi: 10.3989/scimar.03365.03B

Amor MD, Laptikhovsky V, Norman MD, Strugnell JM (2015) Genetic evidence extends the known distribution of Octopus insularis to the mid-Atlantic islands Ascension and St Helena. J Mar Biol Assoc United Kingdom 1–6 . doi: 10.1017/S0025315415000958

Amor MD, Norman MD, Cameron HE, Strugnell JM (2014) Allopatric Speciation within a Cryptic Species Complex of Australasian Octopuses. PLoS One 9: . doi: 10.1371/journal.pone.0098982

Amor MD, Norman MD, Roura A, et al (2016) Morphological assessment of the Octopus vulgaris species complex evaluated in light of molecular-based phylogenetic inferences. Zool Scr 46:275–288 . doi: 10.1111/zsc.12207

Carlini DB, Graves JE (1999) Phylogenetic analysis of cytochrome c oxidase I sequences to determine higher-level relationships within the coleoid cephalopods. Bull Mar Sci 64:57–76

Carlini DB, Young RE, Vecchione M (2001) A Molecular Phylogeny of the Octopoda (Mollusca : Cephalopoda) Evaluated in Light of Morphological Evidence. Mol Phylogenet Evol 21:388–397 . doi: 10.1006/mpev.2001.1022

Dai L, Zheng X, Kong L, Li Q (2012) DNA barcoding analysis of Coleoidea (Mollusca: Cephalopoda) from Chinese waters. Mol Ecol Resour 12:437–447 . doi: 10.1111/j.1755-0998.2012.03118.x

De Luna Sales JB, Do Rego PS, Hilsdorf AWS, et al (2013) Phylogeographical Features of Octopus vulgaris and Octopus insularis in the Southeastern Atlantic Based on the Analysis of Mitochondrial Markers. J Shellfish Res 32:325–339 . doi: 10.2983/035.032.0211

González-Gómez R, Barriga-Sosa IDLA, Pliego-Cárdenas R, et al (2018) An integrative taxonomic approach reveals Octopus insularis as the dominant species in the Veracruz Reef System (southwestern Gulf of Mexico). PeerJ 6:1–30 . doi: 10.7717/peerj.6015

Guerra Á, Roura Á, González ÁF, et al (2010) Morphological and genetic evidence that Octopus vulgaris Cuvier, 1797 inhabits Amsterdam and Saint Paul Islands (southern Indian Ocean). ICES J Mar Sci 67:1401–1407 . doi: 10.1093/icesjms/fsq040

Huffard CL, Saarman N, Hamilton H, Simison B (2010) The evolution of conspicuous facultative mimicry in octopuses: an example of secondary adaptation? Biol J Linn Soc 101:68–77 . doi: 10.1111/j.1095-8312.2010.01484.x

Kaneko N, Kubodera T, Iguchis A (2011) Taxonomic Study of Shallow-Water Octopuses (Cephalopoda: Octopodidae) in Japan and Adjacent Waters using Mitochondrial Genes with Perspectives on Octopus DNA Barcoding. Malacologia 54(2). doi: 10.4002/040.054.0102

Keskin E, Atar HH (2011) Genetic divergence of Octopus vulgaris species in the eastern Mediterranean. Biochem Syst Ecol 39:277–282 . doi: 10.1016/j.bse.2011.08.015

Pliego-Cárdenas R, Flores L, Markaida U, et al (2016) Genetic evidence of the presence of Octopus mimus in the artisanal fisheries of octopus in Santa Elena Peninsula, Ecuador. Am Malacol Bull 34:51–55 . doi: http://dx.doi.org/10.4003/006.034.0102

Pliego-Cárdenas R, Hochberg FG, García de León FJ, Barriga-Sosa IDLA (2014) Close Genetic Relationships between Two American Octopuses: Octopus hubbsorum Berry, 1953, and Octopus mimus Gould, 1852. J Shellfish Res 33:293–303 . doi: 10.2983/035.033.0128

Strugnell J, Norman M, Drummond AJ (2004) Neotenous origins for pelagic octopuses. Curr Biol 14:300–301 . doi: http://dx.doi.org/10.1016/j.cub.2004.03.048

Strugnell J, Norman M, Jackson J, et al (2005) Molecular phylogeny of coleoid cephalopods (Mollusca:Cephalopoda) using a multigene approach; the effect of data partitioning on resolving phylogenies in a Bayesian framework. Mol Phylogenet Evol 37:426–441 . doi: 10.1016/j.ympev.2005.03.020

Strugnell JM, Norman MD, Vecchione M, et al (2014) The ink sac clouds octopod evolutionary history. Hydrobiologia 725:215–235 . doi: 10.1007/s10750-013-1517-6

Takumiya M, Kobayashi M, Tsuneki K, Furuya H (2005) Phylogenetic Relationships among Major Species of Japanese Coleoid Cephalopods (Mollusca : Cephalopoda) Using Three Mitochondrial DNA Sequences. Zoolog Sci 22:147–155

Teske PR, Oosthuizen A, Papadopoulos I, Barker NP (2007) Phylogeographic structure of Octopus vulgaris in South Africa revisited: identification of a second lineage near Durban harbour. Mar Biol 151:2119–2122 . doi: 10.1007/s00227-007-0644-x

Undheim EAB, Norman JA, Thoen HH, Fry BG (2010) Genetic identification of Southern Ocean octopod samples using mtCOI. Comptes Rendus Biologies 333(5): 395-404. doi: 10.1016/j.crvi.2010.02.002
